# Is human life worth peanuts? Risk attitude changes in accordance with varying stakes

**DOI:** 10.1371/journal.pone.0201547

**Published:** 2018-08-09

**Authors:** Kazumi Shimizu, Daisuke Udagawa

**Affiliations:** 1 Department of Political Science and Economics, Waseda University, Shinjukuku, Tokyo, Japan; 2 Faculty of Economics, Hannan University, Amamihigashi Matsubara, Osaka Japan; Technion Israel Institute of Technology, ISRAEL

## Abstract

*Risk aversion* is well-known as a general and robust characteristic of people’s decision making: people are less likely to gamble when they are unsure if they will obtain the expected value of the bet made. The “*peanuts effect*” is, however, an exception to this general rule. The “peanuts effect,” which states that people are more willing to gamble when playing for “peanuts” (a small outcome), has been stably observed in the context of a small monetary stake. We conducted two types of experiments to verify whether the peanuts effect still occurred when the type of stakes changed. We had two main findings. On the one hand, people tended to gamble more for a qualitatively smaller value when the stake was material in nature, meaning that the “*peanuts effect*” occurred with a qualitatively low stake. On the other hand, people were willing to take a risk for a qualitatively larger value when the stake was a human life: this is the opposite phenomenon of the “*peanuts effect*.”

## Introduction

*Risk aversion* is well-known as a general and robust characteristic of people’s decision making: people are less likely to gamble when they are unsure if they will obtain the expected value of the bet made. The “*peanuts effect*,” which was first noted by Markowitz [1], is, however, an exception to this general rule: people are more willing to gamble when playing for “peanuts” (small monetary amounts). People might choose to take a $100 certain gamble over a 10% chance at winning $1,000, but they might prefer to take the 10% chance at winning $10 over receiving $1 for sure.

Although Markowitz himself did not examine this effect experimentally, a number of subsequent studies showed that the peanuts effect remains stable in the context of a monetary stake ([2–5]). In their meta-analysis of published studies on save-or-spoil situations, Kuhberger, Schulte-Mecklenbeck, and Perner [6] stated that higher payoffs usually led to increasing risk aversion when not only money/property but also nonmonetary goods, like jobs or time, are at stake. Moreover studies, presented in the next paragraph, have revealed that a similar phenomenon to the peanuts effect occurred when a human life, which is intuitively qualitatively different from money or other material goods, was at stake.

Table 1 summarizes Wang and colleagues’ research, which shows how people’s attitude to risk changes when contextual group size is manipulated in a life-or-death decision situation. Generally speaking, the results showed that people’s risk-seeking tendency was greater in the context of both positive and negative frames when the affected group was small (e.g., six or 60 people) than when it was large (e.g., 600 or 6,000 people).

**Table 1:** Group size effects: Percentages of participants choosing the probabilistic alternative.

	Wang and Johnston[24]			
	Group size = 6000	Group size = 600	Group size = 60	Group size = 6
Positive frame	40.9% (*n* = 44)	40.0% (*n* = 50)	67.5% (*n* = 40)	64.0% (*n* = 50)
Negative frame	61.4% (*n* = 44)	68.0% (*n* = 50)	65.0% (*n* = 40)	70.0% (*n* = 50)
Framing effects	Yes	Yes	No	No
	Wang[23]			
	Group size = 6000	Group size = 600	Group size = 60	Group size = 6
Positive frame	38.7% (*n* = 31)	41.9% (*n* = 31)	57.6% (*n* = 33)	66.7% (*n* = 30)
Negative frame	66.3% (*n* = 30)	76.5% (*n* = 34)	66.7% (*n* = 30)	75.6% (*n* = 33)
Framing effects	Yes	Yes	No	No
	Wang et al.[25]			
	Group size = 6 billon			Group size = 6
Positive frame	36.0% (*n* = 50)			70.0% (*n* = 50)
Negative frame	66.0% (*n* = 50)			70.0% (*n* = 50)
Framing effects	Yes			No

In accordance with these studies, which mainly used American or European university student samples, our studies of the Japanese general public (e.g., [7–8]) showed the same phenomenon (see Table 2).

**Table 2:** Percentages of the probabilistic choice in the Life-Death decision problem across three sizes in a national survey (N = 966).

	Shimizu and Udagawa [7–8]		
	Group size = 6000	Group size = 60	Group size = 6
Positive frame	31.2% (*n* = 173)	32.6% (*n* = 172)	43.4% (*n* = 166)
Negative frame	45.5% (*n* = 156)	58.4% (*n* = 149)	54.0% (*n* = 150)
Framing effects	Yes	No	No

Smaller group sizes leading to increased risk seeking can be interpreted as a form of the peanuts effect. However, there are two reasons to doubt this interpretation, the first of which is related to differences regarding the degree of risk seeking. In the experiments which stake is human life, people take more risk than in other similar experiments which stake is monetary outcome; for example, Weber and Chapman [9] showed that ratio of subjects choosing the probabilistic option is inferior to one quarter. In contrast, Table 1 above reveals that people exhibited more risk seeking in small-group contexts, e.g., six or 60 people, where more than 60% of the participants chose a gambling alternative. Table 2 reveals that the percentage of Japanese subjects who choose the probabilistic choice remains higher than that reported by usual peanuts effect studies using a monetary account as the stake. This is consistent with the results of Fagley and Miller [10], showing that for outcomes involving human lives rather than money, subjects were more likely to take a chance when the stake size was very large, e.g., between 600/36,000/216,000 people and 600/36,000/216,000 dollars. They suggested that “choice behavior involving human life outcomes in the positive frame is *qualitatively* different from the monetary arena” (p. 369).

The second, and more substantive reason, reflects possible differences in psychological motives. Weber and Chapman[9] suggested based on their experimental results that the peanuts effect could be caused by disappointment. *Disappointment*, as an emotion that is experienced when it is perceived that a different state of the world would have produced a better result, can engender the peanuts effect in that people may be willing to gamble when playing for small stakes, because they recognize they will not feel very much disappointed about the outcome if they lose the gamble. In contrast, for large-stakes gambles, where disappointment is much greater, the anticipated negative emotion may drive people to be more risk averse. Intuitively speaking, when we pick a 10% chance of winning $1 over a sure win of $0.10, we can say “Who cares if I lose? It’s only a dime.” However, is this psychological reasoning applicable to people’s risk-seeking tendency in the context of a life-or-death situation when participants are in a small group size? When we give up four lives from among six people, do we still think that it is a good deal? Studies in evolutionary psychology and anthropology [11–20] have suggested that in contrast to the perceived value of a small amount of money, people might intuitively value a small-, rather than large-, sized group.

If human cooperativeness has evolved in a small group context, then it seems reasonable to suppose that a small group size reminds us of collaborative togetherness. If, on the basis of these discussions, we assume that people feel more attachment to a small group than a big one, the “disappointment” explanation of Weber and Chapman [9] can be used to predict that people should seek fewer risks when in a small, rather than big, group context. Existing data, however, have not confirmed this prediction. Thus, we hypothesize that, in contrast to the peanuts effect, people will be more likely to take risks to obtain a greater value when the stake is human lives.

These two points―differences in risk-seeking degree and psychological motive―lead us to think that the phenomenon of smaller group sizes leading to a higher risk-seeking attitude does not indicate the peanuts effect, in spite of their seeming similarity, because when a human life is at stake people may take more of a risk to obtain a greater value, whereas in the peanuts effect, people are more likely to gamble for a smaller value. Are these two phenomena essentially different? If so, how can we accommodate the difference? The main purpose of this study is to answer these questions by using an experimental method to examine the substantial difference in monetary and human life outcomes on people’s decision making.

## Hypotheses and predictions

This study involved two types of gambling experiments: “life-or-death” and “goods.” The “goods” type also had two subtypes: “drink” and “commodity.” The first one is designed to verify if people really choose to gamble for a greater value when the stakes are human lives, while second one is used to examine if the peanuts effect can be replicated with a material goods, qualitatively evaluated. The first experiment (“life-or-death”) is our primary area of interest, but the second (“drink” and “commodity”) is also important because although many studies examining the peanuts effect have used a monetary stake or other ordinary goods with a money-related value, little is known about whether the peanuts effect occurs with ordinary goods that vary in quality (i.e., “cheap wine” instead of “wine costing $4”). If the peanuts effect does not occur in the context of both material goods (measured qualitatively) and human lives, then this may suggest that the effect is more closely related to quantitative, rather than qualitative, outcomes. By contrast, if the peanuts effect occurs in a quality context but not in a human life context, then this may highlight a substantive difference between the effect of human lives and standard goods on people’s decision making. For both experiments, we principally used the same experimental design as the life-or-death situation developed by Kahneman and Tversky [21], apart from the number of people or items and the quality of stakes.

Following the results of previous research (e.g., [7–8], [22–25]) and the argument in the Introduction, our predictions for the “life-or-death” experiment were as follows.

Prediction 1: Participants will be more likely to be take risks to obtain a greater value when the stake is human lives. Participants will take small risks for people in general (six people condition), more risks for friends (six friends condition), and the most risks for family (family of six condition).
Prediction 2: Participants will be more likely to take chances in small (group of 6 persons) compared to large (group of 600 persons) group size contexts.

Based on previous research regarding the “*peanuts effect*,” we proposed a third prediction for the “goods” experiment.

Prediction 3: Participants will display more risk-seeking for cheap drinks and commodities than for those of high quality.

## Material and methods

The Waseda University Ethical Review Board specifically approved this study.

### Subjects and procedure

To conduct the “life-or-death” and “drink” experiments, the private research company Nikkei Research Inc. was used to recruit subjects. For the “commodity” experiment, the private research company Rakuten Research Inc. was used to recruit subjects. The three experiments were web-based. These subjects had voluntarily applied for membership to the research companies and could choose to answer survey questions via the Internet in their homes, because the experimental instructions were presented on their computer. After the experiment, the company randomly chose some of the respondents and paid them a fee of \500 (approximately US$5–6). The “life-or-death” and “drink” experiments took place from February 18–23, 2011 with 1,049 subjects (483 females and 566 males). The mean age was 35.9 years (*SD*: 14.7, range: 16–69). The “commodity” experiment took place from May 30 to June 6, 2018 with 1153 subjects (576 females and 577 males). The mean age was 49.9 years (*SD*: 15.7, range: 20–99).

### Design

After reading the brief instructions on the computer screen, subjects answered one of four versions of a life-or-death situation (600 people, six people, six friends, or a family of six). As shown in S1 File, for each of these contextual group sizes the life-or-death decision situation was presented either in terms of saving lives (positive framing) or losing lives (negative framing). The subjects were randomly assigned to one of the eight experimental groups and were unaware of the experimental manipulation. Each version of the life-or-death situation had the same mathematical probability structure, wherein the probability of survival was always one-third. The two options were either a sure cure for one-third of the patient group (Plan A) or a one-third probability of finding a cure for the whole group (Plan B). Each subject saw one version of the life-or-death situation and was asked to rate its attractiveness on a scale ranging from 1 (*highly risk averse*) to 6 (*highly attracted to taking risks*), where higher numbers meant that the probabilistic choice was more attractive. As our interest is in subjects’ attitude direction, a 6-point scale is appropriate [26]. When we focus on the subject’s binary choice, the rating answers were converted to choice responses by determining which option had been given the higher rating, that is, ratings from 1 to 3 were considered as a deterministic choice and ratings from 4 to 6 as a probabilistic choice.

After the life-or-death experiment, through questions about academic grounds and numeracy, subjects entered in the “drink experiment” (see S2 File). They saw one version of the drink situation (six cans of soft drink, six bottles of high quality wine, 600 bottles of low quality wine, or 600 bottles of high quality wine) and were asked to rate its attractiveness on the same 6-point scale that was used in the other experiment. Last, we collected demographic details, including sex, age, marital status, residential status, profession, and annual income.

In the “commodity” experiment that was conducted separately from the “life-or-death” and “drink” experiments, each participant saw one version of the commodity situation (six commodities of high quality, six commodities of low quality, 600 commodities of high quality, or 600 commodities of low quality) and were asked to rate its attractiveness on the same 6-point scale that was used in the other experiments (see S3 File). Lastly, we collected demographic details, including sex, age, marital status, residential status, profession, and annual income.

### Results

#### Life-or-death experiment

**Fig 1:**
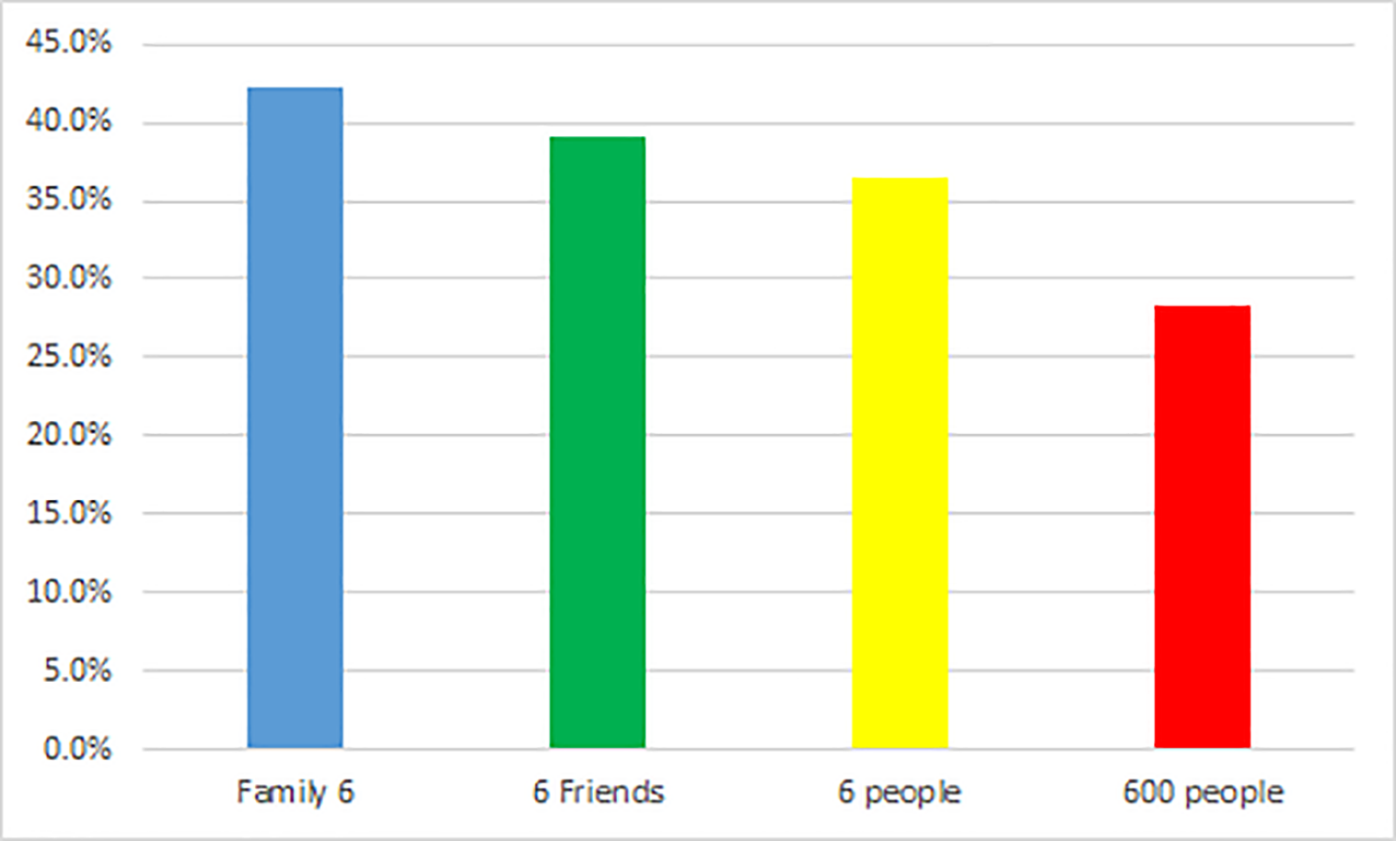
Percentages of risky choice at “Life-Death problem” in the positive frame.

Tables 3 and 4 and Fig 1 show that the peanuts effect did not occur across all three categories, that is, six people, six friends, and a family of six in either framing condition. Subjects exhibited increased willingness to take risks with decreasing group size; the differences in risk-seeking degree between the 600 people context and three groups of six contexts were significant in both framing conditions. The amount of risk the subjects were willing to take increased from six people to six friends and then to family of six; people were willing to make a riskier choice for a greater value.

**Table 3:** Frequencies and percentages of choice in the Life-Death problem across 4 categories (positive frame).

	Plan A is much more attractive than Plan B.	Plan A is more attractive than Plan B.	Plan A is slightly more attractive than Plan B.	Plan B is slightly more attractive than Plan A.	Plan B is more attractive than Plan A.	Plan B is much more attractive than Plan A.	total
Strangers 6	16	26	38	31	9	6	126
	(12.7%)	(20.6%)	(30.2%)	(24.6%)	(7.1%)	(4.8%)	(100.0%)
Friends 6	22	22	34	36	8	6	128
	(17.2%)	(17.2%)	(26.6%)	(28.1%)	(6.3%)	(4.7%)	(100.0%)
Family 6	15	15	45	38	12	5	130
	(11.5%)	(11.5%)	(34.6%)	(29.2%)	(9.2%)	(3.8%)	(100.0%)
Strangers 600	32	26	38	25	6	7	134
	(23.9%)	(19.4%)	(28.4%)	(18.7%)	(4.5%)	(5.2%)	(100.0%)

**Table 4:** Frequencies and percentages of choice in the Life-Death problem across 4 categories (negative frame).

	Plan A is much more attractive than Plan B.	Plan A is more attractive than Plan B.	Plan A is slightly more attractive than Plan B.	Plan B is slightly more attractive than Plan A.	Plan B is more attractive than Plan A.	Plan B is much more attractive than Plan A.	total
Strangers 6	11	24	27	52	11	5	130
	(8.5%)	(18.5%)	(20.8%)	(40.0%)	(8.5%)	(3.8%)	(100.0%)
Friends 6	17	14	38	61	17	6	153
	(11.1%)	(9.2%)	(24.8%)	(39.9%)	(11.1%)	(3.9%)	(100.0%)
Family 6	5	15	29	49	19	7	124
	(4.0%)	(12.1%)	(23.4%)	(39.5%)	(15.3%)	(5.6%)	(100.0%)
Strangers 600	19	17	37	37	12	2	124
	(15.3%)	(13.7%)	(29.8%)	(29.8%)	(9.7%)	(1.6%)	(100.0%)

To closely examine this possible choice reversal, we used the following standard multiple regression model:

$$\begin{array}{l} \mathrm{Attractiveness}\;\mathrm{of}\;\mathrm{the}\;\mathrm{probabilistic}\;\mathrm{choice}=Intercept+\beta_{1}\times Positive\_Frame_{i}\\ \;+\beta_{2}\times 600\_People_{i}\\ \;+\beta_{3}\times Family_{i}\\ \;+\beta_{4}\times Friends_{i}\\ \;+\beta_{5}\times Female_{i}\\ \;+\beta_{6}\times Age_{i} \end{array}$$

In this model, the dependent variable was the attractiveness of the probabilistic choice, which was measured on a scale ranging from 1 (*highly risk averse*) to 6 (*highly attracted to taking risks*). As regards the independent variables, *Positive_Frame* was dummy variable coded as 1 if the participant answered the positive framed situation or 0 for the negative framed situation; *600_People* was dummy variable coded as 1 if the participant answered the 600 situation or 0 for all other contexts. *Family* was dummy variable coded as 1 if the participant answered the family of six situation or 0 for all other contexts; *Friends* was dummy variable coded as 1 if the participant answered the six friends situation or 0 for all other contexts. In this model, regarding the three group dummy variables, the baseline was participants who answered the six people situation. As for the control variables, gender and age were controlled (*Female* was dummy coded as 1 if the participant was female, otherwise 0). Table 5 shows the estimation of this model along with the results of the “life-or-death” experiment.

**Table 5:** Estimates for multiple regression model with 6 response categories.

Independent variables	Coefficients	
*Positive*	-0.358	***
	(0.079)	
*600_People*	-0.264	*
	0.113	
*Family*	0.263	*
	(0.113)	
*Friend*	0.034	
	(0.110)	
*Female*	0.087	
	(0.080)	
*Age*	0.000	
	(0.003)	
(Intercept)	3.352	***
	(0.142)	
df	1042	

Signif. codes

‘***’ 0.001

‘**’ 0.01

‘*’ 0.05

Following this procedure, a positive value of the coefficient of *Family* (*p* < .05) indicated that the participants were significantly more risk seeking in the family of six than in the six people situation. Further, the difference in risk-seeking degree between the six friends and family of six situations was significant at the 5% level (*p* = .039). However, the difference in risk-seeking degree between the six people and six friends situations was non-significant (*p* = .756), although the positive direction of the related coefficient (β_4_) was consistent with our expectation. Overall, participants, attributing the highest value to family, were most likely to take a chance, which is entirely contrary to the peanuts effect. We may, therefore, conclude that Predictions 1 and 2 were upheld, except for the difference between the six friends and family of six contexts.

#### Drink experiment

**Fig 2:**
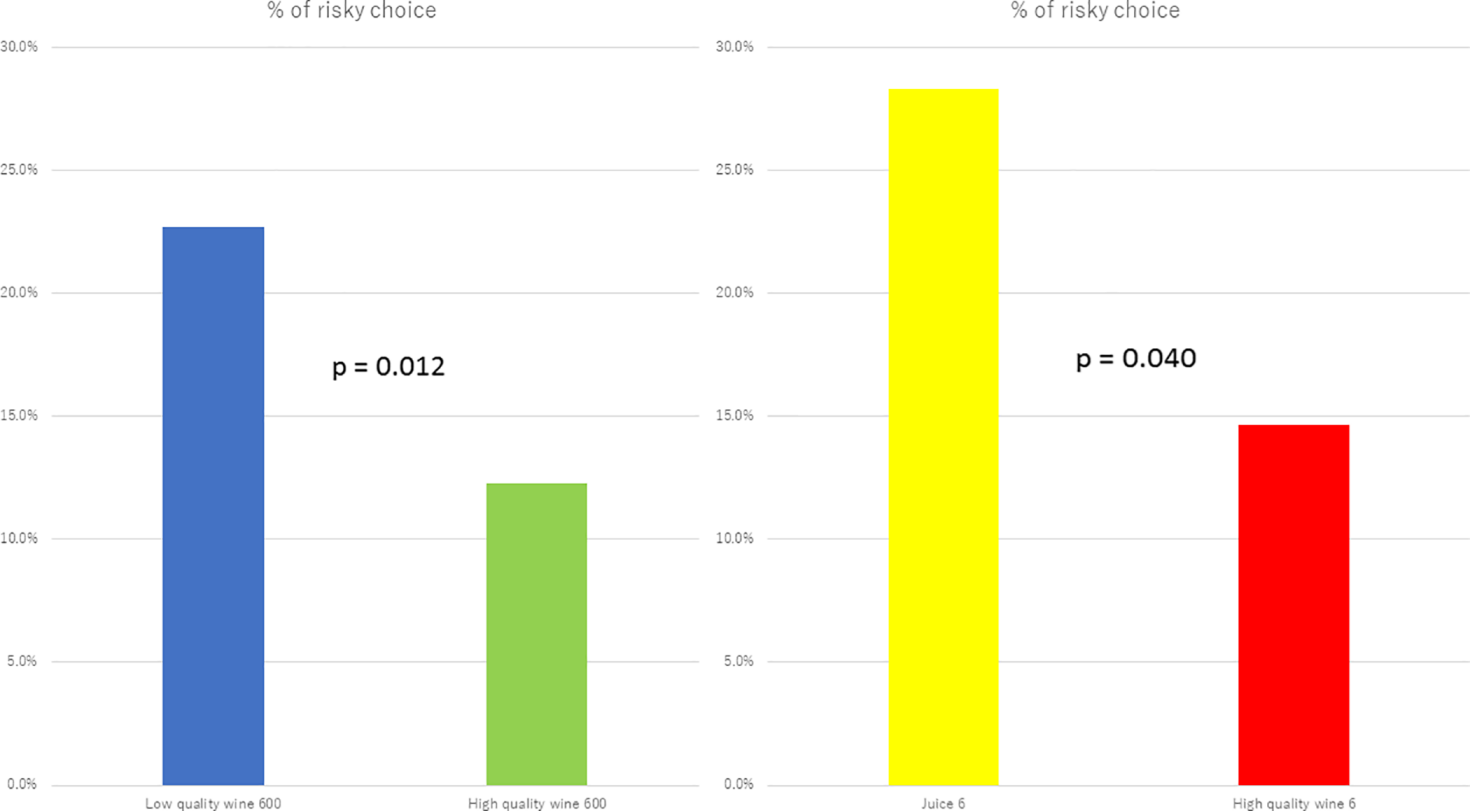
Percentages of risky choice at “Drink problem” in the positive frame without minority.

Tables 6 and 7 and Fig 2 reveal that the peanuts effect was observed in the drink experiment (which measured quality) when subjects were assigned to the positive framing condition; however, in the negative framing condition, the peanuts effect did not occur. This is likely because the framing effect was so strong that there was a ceiling effect of risk seeking. As the peanuts effect should be examined in a positive framing context, we can disregard this nonoccurrence and state that the peanuts effect exists not only in a quantitatively less valuable condition but also in a qualitatively less valuable condition.

**Table 6:** Frequencies and percentages of choice in the Drink problem across 4 categories without minority (positive frame).

	Plan A is much more attractive than Plan B.	Plan A is more attractive than Plan B.	Plan A is slightly more attractive than Plan B.	Plan B is slightly more attractive than Plan A.	Plan B is more attractive than Plan A.	Plan B is much more attractive than Plan A.	total
High quality Wine 6	39	32	34	7	9	2	123
	(31.7%)	(26.0%)	(27.6%)	(5.7%)	(7.3%)	(1.6%)	(100.0%)
Juice 6	36	24	26	23	7	4	120
	(30.0%)	(20.0%)	(21.7%)	(19.2%)	(5.8%)	(3.3%)	(100.0%)
High quality Wine 600	28	47	25	10	2	2	114
	(24.6%)	(41.2%)	(21.9%)	(8.8%)	(1.8%)	(1.8%)	(100.0%)
Low quality Wine 600	38	24	30	17	6	4	119
	(31.9%)	(20.2%)	(25.2%)	(14.3%)	(5.0%)	(3.4%)	(100.0%)

**Table 7:** Frequencies and percentages of choice in the Drink problem across 4 categories without minority (negative frame).

	Plan A is much more attractive than Plan B.	Plan A is more attractive than Plan B.	Plan A is slightly more attractive than Plan B.	Plan B is slightly more attractive than Plan A.	Plan B is more attractive than Plan A.	Plan B is much more attractive than Plan A.	total
High quality Wine 6	11	29	34	22	10	8	114
	(9.6%)	(25.4%)	(29.8%)	(19.3%)	(8.8%)	(7.0%)	(100.0%)
Juice 6	25	12	30	29	14	6	116
	(21.6%)	(10.3%)	(25.9%)	(25.0%)	(12.1%)	(5.2%)	(100.0%)
High quality Wine 600	12	23	47	25	12	2	121
	(9.9%)	(19.0%)	(38.8%)	(20.7%)	(9.9%)	(1.7%)	(100.0%)
Low quality Wine 600	28	20	38	31	17	4	138
	(20.3%)	(14.5%)	(27.5%)	(22.5%)	(12.3%)	(2.9%)	(100.0%)

It is worth noting that the peanuts effect was not observed between “High quality wine 600” and “High quality wine 6,” with a difference of 2.2% (= 14.6%-12.4%). This was probably because the term “High quality” may cover a wide range of values: “High quality wine 600” was not necessarily more valuable than “High quality wine 6” unless “High quality” could be standardized in some way such as in terms of a monetary unit.

#### Commodity experiment

Table 8 shows that the peanuts effect is not evident when comparing high quality commodity 6 versus low quality commodity 6 (*p* = .075), and that the effect does not exist when comparing high quality commodity 600 versus low quality commodity 600 (*p* = .578). This non-occurrence may be due to the ambiguity of the term “commodity.” For example, if a participant imagined a car as a commodity, 6 or 600 cars of low quality would not be considered to be peanuts. In other words, the term “commodity” did not allow sufficient control over a participant’s perception of the stake in the experiment. Consistent with the argument above, if we compare the low quality commodity 6 context and the high quality commodity 600 context, possibly the largest difference of values, we observe in the positive frame that participants were more likely to gamble in the former than in the latter condition (*p* = .042). This suggests that the peanuts effect may occur in a qualitative context if the researcher can control the value of the stake. In addition, Table 9 reveals that in the negative framing condition, the peanuts effect does not occur as well as in the drink experiment.

Based on the results of the drink and commodity experiments, we conclude that while Prediction 3 holds when the stake is appropriately defined, the prediction does not hold when the stake is abstractly described.

**Table 8:** Frequencies and percentages of choice in the commodity problem across 4 categories (positive frame).

	Plan A is much more attractive than Plan B.	Plan A is more attractive than Plan B.	Plan A is slightly more attractive than Plan B.	Plan B is slightly more attractive than Plan A.	Plan B is more attractive than Plan A.	Plan B is much more attractive than Plan A.	total
High quality Commodity 6	6	21	13	8	4	1	53
	(11.3%)	(39.6%)	(24.5%)	(15.1%)	(7.5%)	(1.9%)	(100.0%)
Low quality Commodity 6	8	14	14	12	11	2	61
	(13.1%)	(23.0%)	(23.0%)	(19.7%)	(18.0%)	(3.3%)	(100.0%)
High quality Commodity 600	11	19	15	5	9	3	62
	(17.7%)	(30.6%)	(24.2%)	(8.1%)	(14.5%)	(4.8%)	(100.0%)
Low quality Commodity 600	8	16	11	11	4	3	53
	(15.1%)	(30.2%)	(20.8%)	(20.8%)	(7.5%)	(5.7%)	(100.0%)

**Table 9:** Frequencies and percentages of choice in the commodity problem across 4 categories (negative frame).

	Plan A is much more attractive than Plan B.	Plan A is more attractive than Plan B.	Plan A is slightly more attractive than Plan B.	Plan B is slightly more attractive than Plan A.	Plan B is more attractive than Plan A.	Plan B is much more attractive than Plan A.	total
High quality Commodity 6	8	23	15	7	2	2	57
	(14.0%)	(40.4%)	(26.3%)	(12.3%)	(3.5%)	(3.5%)	(100.0%)
Low quality Commodity 6	14	19	13	7	5	5	63
	(22.2%)	(30.2%)	(20.6%)	(11.1%)	(7.9%)	(7.9%)	(100.0%)
High quality Commodity 600	21	22	9	6	0	1	59
	(35.6%)	(37.3%)	(15.3%)	(10.2%)	(0.0%)	(1.7%)	(100.0%)
Low quality Commodity 600	11	27	10	3	4	1	56
	(19.6%)	(48.2%)	(17.9%)	(5.4%)	(7.1%)	(1.8%)	(100.0%)

## Discussion and conclusions

Considering the results of previous research on the peanuts effect and those of our experiments above, we can conclude that the decision maker’s risk attitude may vary with the type of stakes: while people tend to be willing to take a risk for a greater value when the stake is human lives, they tend to gamble more (take a higher risk) for a smaller value (either quantitatively nor qualitatively) when the stake is monetary/material in nature. This risk-seeking for human life may be explained by egalitarian motives of human beings.

Anthropological and experimental studies show that humans are willing to allocate their scarce resources to promote equality in groups, from hunter-gatherer societies through to laboratory experiments [27–29]. As group size in these laboratory studies was rarely greater than 10 and the groups of hunter-gatherer societies are comprised of family, we expect egalitarian motives to be most present for our family of 6 condition. If participants chose Plan A in this condition, they allocated the survival probability (a scarce resource) *unequally* to 6 people: 100% survival chance for 2 and 0 for the others. If participants chose Plan B in this condition, they allocated the probability *equally* to all 6: 1/3 chance for all. Provided egalitarian motives were present in the family of 6 condition, they would be more likely to prefer Plan B to A.

If individuals are willing to risk more when the stake is human lives, this bias could have political and economic implications. First, those responsible for human life, such as social planners (governors) or doctors, should know about this bias, as it may unconsciously influence and bias their rational decision making about “who should be saved versus not saved.” Second, choices about the best life insurance plan for your family could be distorted by this bias.

Further investigation is necessary to explore this bias. First, instead of the disappointment explanation, which was given by Weber and Chapman [9], we should clarify the psychological mechanism that makes us willing to gamble for a greater value when the stake is human life. Second, if this mechanism can be identified, we should examine whether it is coherent with our egalitarian motive explanation. After these steps are accomplished, the bias observed in the present study may be explained by egalitarian *moral sentiment*, that follows recent neural studies claiming that *emotional* brain mechanisms underlie egalitarian behavior in humans [30,31].

## Supporting information

S1 File Versions of the decision situation in the life-or-death experiment: Positive framing version. (DOCX)

S2 File Versions of the decision situation in the drink experiment: Positive framing version. (DOCX)

S3 File Versions of the decision situation in the commodity experiment: Positive framing version. (DOCX)
