# Supplementary material for: Is human life worth peanuts? Risk attitude changes in accordance with varying stakes
Source: PLoS One. 2018 Aug 9;13(8):e0201547. doi: 10.1371/journal.pone.0201547 (PMC6084950; doi:10.1371/journal.pone.0201547)
Supplement: S1 File — (DOCX) [file pone.0201547.s001.docx]

**Appendix A: Versions of the Decision Situation in the Life-or-Death Experiment**

**Positive Framing Version**

Imagine that six people (six friends, a family of six, 600 people) are infected by a fatal disease. Two alternative medical plans to treat the disease have been proposed. Assume that the exact scientific estimates of the consequences of the plans are as follows:

If plan A is adopted, two people (two friends, two of family, 200 people) will be saved.

If plan B is adopted, there is a one-third probability that all six people (six friends, family of six, 600 people) will be saved, and a two-thirds probability that none of them will be saved.

To what extent would you prefer each of these plans?

1. I prefer plan A to plan B very strongly.
2. I prefer plan A to plan B strongly.
3. I prefer plan A to plan B somewhat strongly.
4. I prefer plan B to plan A somewhat strongly.
5. I prefer plan B to plan A strongly.
6. I prefer plan B to plan A very strongly.
