# Supplementary material for: Is human life worth peanuts? Risk attitude changes in accordance with varying stakes
Source: PLoS One. 2018 Aug 9;13(8):e0201547. doi: 10.1371/journal.pone.0201547 (PMC6084950; doi:10.1371/journal.pone.0201547)
Supplement: S2 File — (DOCX) [file pone.0201547.s002.docx]

**Appendix B: Versions of the Drink Decision Situation in the Drink Experiment**

**Positive Framing Version**

Imagine that in a warehouse there are six cans of juice (six bottles of high quality wine, 600 bottles of cheap wine, 600 bottles of high quality wine) that will be totally spoiled without prompt remedy. Two alternative remedy plans to treat this situation have been proposed. Assume that the exact scientific estimates of the consequences of the plans are as follows:

If plan A is adopted, two cans of juice (two bottles of high quality wine, 200 bottles of cheap wine, 200 bottles of high quality wine) will be saved.

If plan B is adopted, there is a one-third probability that all six cans of juices (six bottles of high quality wine, 600 bottles of cheap wine, 600 bottles of high quality wine) will be saved, and a two-thirds probability that none of them will be saved.

To what extent would you prefer each of these plans?

1. I prefer plan A to plan B very strongly.
2. I prefer plan A to plan B strongly.
3. I prefer plan A to plan B somewhat strongly.
4. I prefer plan B to plan A somewhat strongly.
5. I prefer plan B to plan A strongly.
6. I prefer plan B to plan A very strongly.
